# Paths of lateral gene transfer of lysyl-aminoacyl-tRNA synthetases with a unique evolutionary transition stage of prokaryotes coding for class I and II varieties by the same organisms

**DOI:** 10.1186/1471-2148-6-22

**Published:** 2006-03-12

**Authors:** Shaul Shaul, Ruth Nussinov, Tal Pupko

**Affiliations:** 1 Department of Zoology, George S. Wise Faculty of Life Sciences, Tel Aviv University, Ramat Aviv 69978, Israel; 2 Basic Research Program, SAIC-Frederick, Inc. Center for Cancer Research, Nanobiology Program, NCI-Frederick Frederick, MD 21702, USA; 3 Sackler Inst. of Molecular Medicine, Department of Human Genetics and Molecular Medicine, Sackler School of Medicine, Tel Aviv University, Tel Aviv 69978, Israel; 4 Department of Cell Research and Immunology, George S. Wise Faculty of Life Sciences, Tel Aviv University, Tel Aviv 69978, Israel

## Abstract

**Background:**

While the premise that lateral gene transfer (LGT) is a dominant evolutionary force is still in considerable dispute, the case for widespread LGT in the family of aminoacyl-tRNA synthetases (aaRS) is no longer contentious. aaRSs are ancient enzymes, guarding the fidelity of the genetic code. They are clustered in two structurally unrelated classes. Only lysine aminoacyl-tRNA synthetase (LysRS) is found both as a class 1 and a class 2 enzyme (LysRS1-2). Remarkably, in several extant prokaryotes both classes of the enzyme coexist, a unique phenomenon that has yet to receive its due attention.

**Results:**

We applied a phylogenetic approach for determining the extent and origin of LGT in prokaryotic LysRS. Reconstructing species trees for Archaea and Bacteria, and inferring that their last common ancestors encoded LysRS1 and LysRS2, respectively, we studied the gains and losses of both classes. A complex pattern of LGT events emerged. In specific groups of organisms LysRS1 was replaced by LysRS2 (and vice versa). In one occasion, within the alpha proteobacteria, a LysRS2 to LysRS1 LGT was followed by reversal to LysRS2. After establishing the most likely LGT paths, we studied the possible origins of the laterally transferred genes. To this end, we reconstructed LysRS gene trees and evaluated the likely origins of the laterally transferred genes. While the sources of LysRS1 LGTs were readily identified, those for LysRS2 remain, for now, uncertain. The replacement of one LysRS by another apparently transits through a stage simultaneously coding for both synthetases, probably conferring a selective advantage to the affected organisms.

**Conclusion:**

The family of LysRSs features complex LGT events. The currently available data were sufficient for identifying unambiguously the origins of LysRS1 but not of LysRS2 gene transfers. A selective advantage is suggested to organisms encoding simultaneously LysRS1-2.

## Background

In protein synthesis the rules of the genetic code are established through catalytic aminoacylation of tRNAs by their cognate synthetases. With some notable exceptions, each aaRS enzyme acylates a specific amino acid to its cognate tRNA. Throughout the three Domains of life synthetases are partitioned into two structurally and evolutionary unrelated classes (class 1 and class 2 aaRS) [1]. These differ in their secondary structure arrangements, in their conserved sequence motifs composing the active site, and in the side of the tRNA acceptor stem to which they dock [2]. LysRS is the only known exception to this classification, aminoacylating tRNA(Lys) by two rather than by one enzyme: LysRS1 featuring the distinct structure and characteristics of a class 1 aaRS and LysRS2 with the distinct structure and characteristics of a class 2 aaRS [3]. Structural studies of LysRS1 and LysRS2 complexed with lysine indicated that in addition to the canonical aaRS class distinctions, the amino acid binding site of LysRS1 is more compact than that of LysRS2 [4,5].

All known Eukaryotae apparently code only for LysRS2. Most Bacteria code for LysRS2, but some taxa, predominantly within the class of alpha-proteobacteria, code for LysRS1. Archaea mostly code for LysRS1, with some exceptions coding for LysRS2. R.F. Doolittle and J. Handy [6] predicted that prokaryotes will be found coding for both types of LysRS by the same organism. For a while this prediction was not accepted [7,8]. Recently, it was confirmed: the genomes of several mesophilic prokaryotes were shown to encode both LysRS1 (*lysK*) and LysRS2 *(lysS*). Already five such organisms have been identified: *Methanosarcina mazei *[9], *Methanosarcina acetivorans *[10], *Methanosarcina barkeri *[11], *Bacillus cereus *[12] and *Treponema palladium *[13].

With continuing increase of complete genome sequencing, there is no compelling reason to doubt that the number of prokaryotae discovered to code for both classes of LysRS will rise. The growing database of LysRS1 and LysRS2 synthesized by various archaeal and bacterial phyla and sometimes by the same organism motivated us to address two issues: (1) what are the incidences, patterns and sources of LysRS1 and LysRS2 LGTs between prokaryotes found in current databases of completely sequenced genomes; (2) what is the likely explanation for the phenomenon of organisms retaining both classes of LysRS? To clarify these issues, we reconstructed the relevant archaeal and bacterial species trees, made the most parsimonious assignments of LysRS classes to the ancestral nodes of the trees, reconstructed LysRS1-2 gene trees in order to determine the probable origins of the transferred genes, reviewed the literature for organisms encoding simultaneously two varieties of aaRSs, albeit of the same class, and evaluated the significance of the experimentally determined distinction between the amino acid binding sites of LysRS1-2 in the context of the phenomenon of Archaea and Bacteria encoding both classes of synthetases.

Our analysis of the collected data confirmed that the extant distribution of LysRS1 and LysRS2 reflects a wide-spread LGT – characteristic for the entire aaRS family of enzymes [14,15]. It enabled us to determine some of the most likely paths and several of the origins of these LGT events, and to elucidate the probable selective advantage to several prokaryotes encoding simultaneously both enzyme classes in the presence of environmentally dependent LysRS inhibitors.

## Results

### The evolutionary position of organisms coding for LysRS1, LysRS2 and both enzymes simultaneously

**Figure 1:**
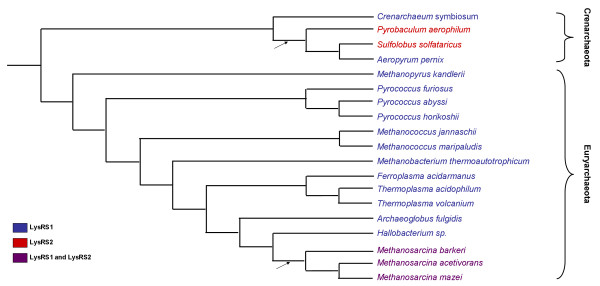
Archaeal tree. Species tree for 19 Archaea encoding LysRS1 and LysRS2. Organisms coding for LysRS1 are colored blue, those coding for LysRS2 are in red, and the three Archaea that code both are in purple. Arrows indicate inferred LGT events.

**Figure 2:**
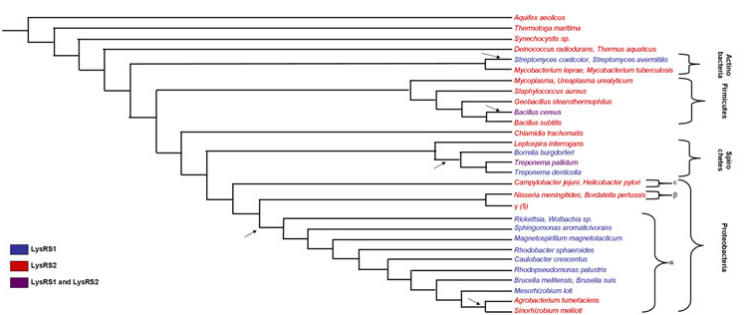
Bacterial tree. Species tree for 43 Bacteria encoding LysRS2 and LysRS1. *Mycoplasma* in the figure refers to both *Mycoplasma pneumoniae *and *Mycoplasma genitalium*. *Gamma-proteobacteria (5)* refers to the following five species: *Escherichia coli*, *Haemophilus influenzae, Buchnera aphidicola, Coxiella burnetii*, *Salmonella typhimurium. Rickettsia *refers to both *Rickettsia conorii a*nd *Rickettsia prowazekii *Organisms coding for LysRS1 are colored blue, those coding for LysRS2 are in red, and the two Bacteria that code both are in purple. Arrows indicate inferred LGT events.

Except for the Archaeon *Cenarchaeum symbiosum* whose genome is still being sequenced, all organisms analyzed in this study for the reconstruction of the species trees have had their entire genomes sequenced and annotated. Thus, there is a reliable assignment for each organism whether it codes for LysRS1 and/or LysRS2. The assignments for Archaea and Bacteria are presented in figs. 1 and 2 respectively. The occurrences of LGT events during the evolution of both Bacteria and Archaea are evident from the fact that both LysRSs are found in the two Domains (figs 1, 2).

### LGTs in Archaea and the LysRS of the last common ancestor of Archaea

The most parsimonious assignment of LysRS types to the ancestral nodes in fig. 1 requires a minimum of two LGT events (marked by arrows in the figure). The majority of Archaea only code for LysRS1. Indeed, according to the most parsimonious reconstruction, their last common ancestor coded for LysRS1. The alternative scenario, in which the ancestor codes for LysRS2, requires two additional LGT events (fig. 1). The three Methanosarcinales – *M. barkeri, M. acetivorans*, and *M. mazei *– code for both enzyme classes. Thus, it seems that their last common ancestor received the gene for LysRS2 via LGT. Since they are monophyletic, only a single LGT is needed to explain the presence of both enzyme classes in this group.

The situation is more complex in Crenarchaeota (fig. 1). In this clade, two families code for LysRS1 (*C. symbiosum *and *Aeropyrum pernix*), and two for LysRS2 (*Pyrobaculum aerophilum *and *Sulfolobus solfataricus*). There are several possible scenarios that can explain this phenomenon:

1. The common ancestor of *P. aerophilum, S. solfataricus *and *A. pernix *first received a copy of LysRS2, and then lost its copy of LysRS1. Subsequently, *A. pernix *received a copy of LysRS1, and lost its copy of LysRS2 (two gains and two losses).

*2. P. aerophilum *after its divergence, gained a copy of LysRS2, and lost its copy of LysRS1. Similarly, after its divergence *S. solfataricus *gained a copy of LysRS2, and lost its copy of LysRS1 (two gains and two losses).

3. The common ancestor of *P. aerophilum, S. solfataticus *and *A. pernix *received a copy of LysRS2. After the divergence of *P. aerophilum*, this organism lost its class 1 copy. The same loss occurred again, after the divergence of *S. solfataricus*. A third loss, of class 2 occurred in the lineage leading to *A. pernix*. This alternative requires one gain, and three losses.

Among these three scenarios, the third requires the least number of LGTs. When an organism codes for both classes of LysRS, a loss event of one copy may be sustainable. Assuming that a loss of one copy out of two is more likely than a LGT, the third scenario is the most probable one. It should be noted that all these alternatives rely on the correctness of the species tree. However, the phylogeny among *P. aerophilum, S. solfataricus *and *A. pernix *(fig. 1) was reconstructed with 100% bootstrap support [16]. See section 'Are the species-trees correct?' below for further discussion on the robustness of the species tree.

### LGTs in Bacteria and the LysRS of the last common ancestor of Bacteria

The most parsimonious assignment of LysRS types to the ancestral nodes in fig. 2 requires a minimum of five LGT events (marked by arrows in the figure). The majority of Bacteria only code for LysRS2. It is most parsimonious to assume that the bacterial ancestor coded for LysRS2 (fig. 2). Among Actinobacteria, some species code for LysRS2 (*Mycobacterium leprae *and *Mycobacterium tuberculosis*), and some code for LysRS1 (*Streptomyces coelicolor *and *Streptomyces avermitilis*). The two species coding for LysRS1 are monophyletic [17]. Thus, it seems that a LysRS1 was laterally transferred to the common ancestor of *Streptomycetes *followed by a LysRS2 loss. Within the Firmicutes, *B. cereus *codes for both LysRS1 and LysRS2. Since this is the only known Firmicutae that codes for both types, we conclude that the LysRS1 was transferred to this species. Within the Spirochetes clade, *Leptospira integrans *codes for LysRS2, while in the second group, including the *T. pallidum*, *Treponema denticola *and *Borrelia burgdorferi *species, the last two species code for LysRS1 only, while *T. pallidum *codes for both LysRS types. This can be explained by a single LGT event (gain of LysRS1) in the common ancestor of *T. pallidum*, *T. denticola*, and *B. burgdorferi*, followed by a LysRS2 loss in *T. denticola *and *B. burgdorferi*. Within the proteobacteria, the beta, gamma, and epsilon clades, all code for LysRS2. Only within the alpha-proteobacteria most species code for LysRS1 (*Rickettsia conorii*, *Rickettsia prowasekii*, *Wolbachia *sp., *Sphingomonas aromaticivorans*, *Magnetospirillum magnetotacticum*, *Rhodobacter sphaeroides*, *Caulobacter crescentus*, *Rhodopseudomonas palustris*, *Brusella malitensis*, *Brucella suis*, and *Mesorhizobium loti*). Following the branching pattern of the Proteobacteria (fig. 2) it seems that the last common ancestor of the alpha-proteobacteria had gained a copy of LysRS1, and lost its LysRS2. However, the clade including the rhizobiales *Sinorhizobium meliloti *and *Agrobacterium tumefaciens *codes for LysRS2. Thus, it seems that in the common ancestor of these species, LysRS2 was regained, and LysRS1 was lost. This scenario calls for two LGT events and two losses.

### Gene trees

Gene trees summarize our current estimation of the evolutionary relationships among the LysRS sequences. Combined with the species tree, gene trees are a valuable source of information concerning the origins of the laterally transferred genes.

### The origin of the inferred LGTs in Archaea

**Figure 3:**
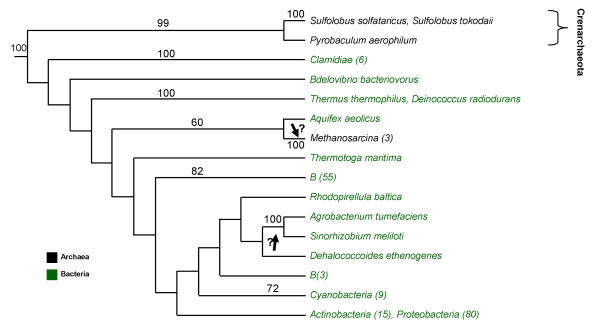
LysRS2 gene tree. Maximum likelihood phylogenetic tree for 177 bacterial and 6 archaeal LysRS2 sequences, colored green and black, respectively. The tree has been rooted using four AspRS sequences (*B. aphidicoda, E. coli, C. jejuni, T. maritima*). Bootstrap percentage values greater than 50% are indicated. Arrows indicate plausible paths for LGT events. Question marks indicate considerable uncertainties as to the origin of the LGT. *Clamidiae (6)* refers to 6 species specified in SM, additional file 1. *Methanosarcina (3)* refers to *Methanosarcina acetivorans, Methanosarcina mazei, Methanosarcina barkeri*. *B (55)* refers to 55 bacterial species: 3 *Clostridia*, 26 *Bacilli*, 13 *Molicutes*, 1 *Fusobacteria*, 2 *Clostridia*, 3 *Bacteroidestes*, 6 *Chlorobia* and 1 *Spirochaetae*. *B (3)* refers to the following 3 bacterial species: *Solibacter usitatus, Anaeromyxobacter dehalogenans, Symbiobacterium thermophilum *The species included in the groups *Cyanobacteria* (9), *Actinobacteria (15) *and *Proteobacteria (80) *are provided as Supplementary Material, additional file 1. Listing of the bacterial and archaeal phyla, classes and species, with corresponding LysRS2 accession numbers and their sources are also provided in SM, additional file 1. The complete (unabbreviated) LysRS2 ML trees are given as SM, additional file 3.

The maximum parsimony analysis has indicated that the ancestor of the Archaea most likely coded for LysRS1. We reconstructed a LysRS2 gene tree (fig. 3) to track the origin of the genes that were laterally transferred to the Archaea. Since our species tree (fig. 2) does not contain all the known LysRS2 sequences, we used blastp [18] to enlarge our bacterial LysRS2 database by choosing the first 177 non-redundant sequences – 131 from complete genomes and 46 encoded by bacteria whose entire genome has not been sequenced yet. AspRS sequences were used to root the tree.

The LysRS2 genes of *P. aerophilum, S. solfataricus*, and *Sulfolobus tokodaii*, cluster together in the gene tree with a very high bootstrap value (99%) supporting our conclusion of a single LysRS2 LGT to the Crenarchaeota clade. This clade has only a low bootstrap value (36%) with respect to genes located on other branches of the tree. Two alternatives can explain such a position: (1) The LGT from Bacteria to Crenarchaeota is from an ancient ancestor of the bacterial Domain or from an extinct bacterial lineage that is an outgroup to most extant Bacteria, or from a yet unidentified bacterium. (2) There is not enough information to resolve the location of this clade within the LysRS2 gene tree, as is evident by the low bootstrap value. In the first alternative, it is not very likely that the LGT is from the bacterial ancestor, as we know that the LysRS2 LGT to Crenarchaeota occurred after the divergence of *C. symbiosum *(fig. 1). All these possibilities are likely scenarios and further bacterial genome sequencing has the potential to settle this issue.

*M. barkeri*, *M. acetivorans *and *M. mazei *code for both classes of LysRS. The origin of their LysRS2 gene is in doubt. The group clusters with the bacterial lineage *A. aquifex *with a low bootstrap value (60%). Again, additional genomic bacterial sequencing might shed light on the history of this LGT event.

### The origin of the inferred LGTs in Bacteria

**Figure 4:**
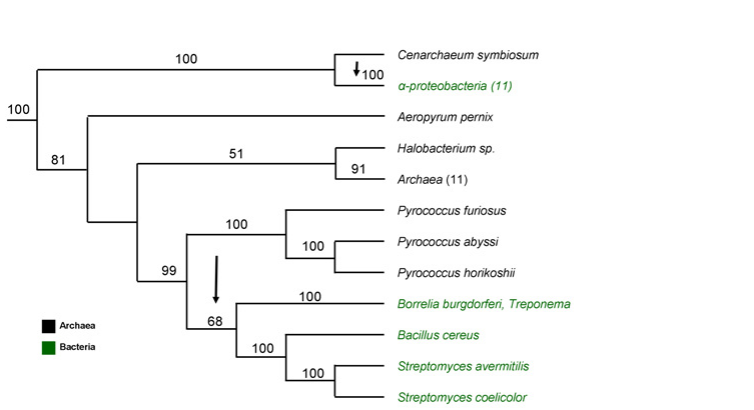
LysRS1 gene tree. Maximum likelihood phylogenetic tree for 17 archaeal and 17 bacterial LysRS1 sequences, colored black and green, respectively. The tree has been rooted using four GluRS sequences (*B. aphidicoda, E. coli, C. jejuni, T. maritima*). Bootstrap percentage values greater than 50% are indicated. Arrows indicate plausible paths for LGT events. *Treponema *refers to *Treponema denticola *and *Treponema pallidum*. The species included in the groups *alpha-proteobacteria (11)*, *Archaea (11)* are provided as SM, additional file 2. Listing of the archaeal and bacterial phyla, classes and species, with corresponding LysRS1 accession numbers and their sources are also provided in additional file 2. The complete (unabbreviated) LysRS1 ML tree is given as SM, additional file 4.

Five LGT events were inferred in the bacterial tree (fig. 2). A gene tree of LysRS1 sequences encoded by Archaea and Bacteria was reconstructed in order to infer the origin of the laterally transferred genes (fig. 4). Several LysRS1 sequences encoded by bacteria whose complete genomes have not been determined yet – e.g., *Bradyrhizobium sp, Rickettsia sibirica, Borella afzelii *– were excluded from this study due to high percentage identity, 61, 88 and 95%, between their sequences and sequences of bacterial LysRS1 utilized for the reconstruction of the gene tree (from *M. loti*, *R. prowazekii *and *B. burgdorferi *respectively). GluRS sequences were used to root the tree. The bacterial sequences of the *S. coelicolor, S. avermitilis, B. cereus, B. burgdorferi, T. pallidum*, and *T. denticola*, together with the archaeal *Thermococcaceae *clade (*P. horikoshii*, *P. abyssi *and *P*. *furiosus*) cluster together with a very high bootstrap support (99%). Such clustering is indicative of a Thermococcaceal source for the LysRS1 found in all bacterial sequences excluding the alpha proteobacteria.

The LysRS1 sequences in alpha proteobacteria cluster with *C. symbiosum *with a very high bootstrap support (100%). Thus, *C. symbiosum *or a related yet undetermined Archaea is the most likely source for the LGT to alpha proteobacteria excluding the two species *A. tumerfaciens*, and *S. meliloti*. Within the alpha proteobacteria, these two species reversed to a LysRS2 class gene. To infer the origin of this reversed LGT, we refer to fig. 3. See Discussion, section 'The possible origins of laterally transferred LysRS genes' below, for details.

## Discussion

### Most likely scenarios for LysRS LGT

In this study we analyzed the pattern of LysRSs LGT based on organisms with fully sequenced genomes, coding for LysRS1, LysRS2, or both. Analyzing this information and taking into account the evolutionary relationships among the organisms (the species tree) made it possible to infer the most likely LGT scenarios. As previously determined by other researchers for the entire family of aaRS enzymes [14], we also found that LGT for a particular synthetase – LysRS – is quite common in both Bacteria and Archaea. Inferring that the last common ancestors of Bacteria and Archaea most likely coded for LysRS2 and LysRS1, respectively, a complex pattern of LGT events emerged: LysRS1 was replaced by LysRS2 (and vice versa) in a specific group of organisms. In one occasion, a LysRS2 to LysRS1 LGT was followed by a reversed LGT to LysRS2, within the same group. It should be noted that a transition from one LysRS to another most probably occurred through an intermediate evolutionary stage in which organisms coded for both LysRSs [15]. Examples of extant species embodying such a stage are the three *Methanosarcinales*, *B. cereus*, and *T. pallidum*

The LysRS1 and LysRS2 genes coded by *T. pallidum *probably illustrate an advanced phase of such a transitional stage: (i) the LysRS2 gene only codes for 351 residues [19]. This region shows a high similarity to the 376 residues of the *E. coli *LysRS2 catalytic domain located in the COOH-terminal region. However, the 144 residues at the NH2-terminal region in the *E. coli *enzyme, which includes the 80 residues of the tRNA (Lys) anticodon binding domain that are critical for the enzyme's acylation activity, are not coded *in T. palladium *[13]. The observed lack of the LysRS2 anticodon binding domain is the result of a LysRS1 gene entering the common ancestor of *T. palladium *and *B. burgdorferi *by LGT from an archaeal lineage [20]. LysRS1 proved by some measure more advantageous to *Treponema *than LysRS2. The latter became non-functional, subject in the course of evolution to gradual elimination from the genome accompanying the loss of function.

### Are the species-trees correct?

Our results depend on inferred species trees that might not be the true ones. Nevertheless, they do not rely on the existence of clades with low statistical support. For the archaeal tree (fig. 1), *M. barkeri*, *M. acetivorans*, and *M. mazei *which code for both LysRS1 and LysRS2 are monophyletic [21]. Further, their clustering with *Hallobacterium *is supported with high bootstrap values [22]. The phylogenetic position of *P. aerophilum, S. solfataricus *and *A. pernix *is also generally accepted [16]. For the bacterial tree (fig. 2), there is wide agreement regarding the monophyly of *alpha-proteobacteria *and the monophyly of *Spirochetes *[23].

### The possible origins of laterally transferred LysRS genes

We determined seven LGT events – two in Archaea and five in Bacteria. One of the main difficulties in the inference of the origins of LGTs is that such inference heavily relies on a gene tree. A gene tree is always reconstructed from a single gene, and hence, based on a limited amount of data. Thus, the bootstrap values for various bifurcations in the tree are usually not very high. It is well known that increased taxonomical sampling improves such inference [24]. To this end, we reconstructed the LysRS gene trees from an extensive database of extant Bacteria and Archaea. Not surprisingly, we could not reliably infer the origins of the two LysRS2 LGTs to Archaea (figs. 1 and 3). Encouragingly, the possible origins of four of the bacterial LGTs (*Actinobacteria*, *B*. *cereus*, *alpha-proteobacteria *and *Spirochetae*) were determined with a high degree of confidence (figs. 2 and 4). For example the archeal *Pyrococci *clade seems to contain the closest LysRS1 sequences to those of bacterial species (fig. 4). Yet, the details of the LGT events are still unknown: These species are hyperthermophiles, inhabiting environments with extremely high temperatures such as undersea hot vents, whereas all the above mentioned bacteria are mesophiles. The physiological and biochemical conditions that promoted such an evolutionary event remain an enigma.

The last intriguing question concerns the LGT reversal of two alpha-proteobacterial species (*A. tumefaciens *and *S. meliloti*) to code for LysRS2. The bootstrap value clustering them with other bacteria is very low (39% with *Dehalococcus ethenogenes*, see SM, Additional file 3). As both species are capable of nitrogen-fixing [25,26], we speculate that an extinct nitrogen fixing bacteria may have been the origin of the LysRS2 LGT.

Additional sequences of bacterial LysRS2 genes are likely to shed new light on the evolution of the LysRS2 LGT events for which the origin remains uncertain. It is remarkable that the sources of LysRS1 LGTs are readily identifiable while those for LysRS2 remain, for now, shrouded in uncertainty.

### Possible advantages for organisms coding for both LysRS classes

Long before the discovery of Archaea and Bacteria coding for both LysRSs, it was found that some prokaryotes code for two paralogous genes for some synthetases: *lysS *and *lysU *in *E.coli* 33, *thrSv *and *thrS2 *and *tyrS *and *tyrZ*, in *B. subtilis *[34,35]. Recently, co-existing forms were published for SerRS1/SerRS2 and TrpRS1/TrpRS2 in *C. acetobutylicum*, CysRS1/CysRS2 in *M. tuberculosis*, TrpRS1/TrpRS2 in *E. faecalis *[14] and GluRS1/GluRS2 in more than 30 bacterial genomes [14,27-31]. While only some of the functions of the observed redundancies have been determined, it is noteworthy that in some cases it was found that the aaRS duplications render a selective advantage to the affected organisms providing protection against potentially detrimental effects on protein synthesis caused by amino acid competitors [32].

One example is the *Streptococcus pneumoniae *coding for two distantly related MetRS genes. It was found that one of them proves necessary and sufficient for resistance to MetRS inhibitors [33]. Another example is the existence of two IleRS variants in *Pseudomonas fluorescens. *This gamma proteobacterium produces the anti-bacterial agent pseudomonic acid (mupirocin), which if not neutralized, competitively inhibits the acylation of tRNA(Ile) with isoleucine, thereby shutting off protein synthesis and arresting cell growth. *P. fluorescens *avoids self destruction by one of its IleRS variants binding preferentially to isoleucine, with a remarkably high insensitivity to mupirocin [34].

A related selective advantage is surmised for the prokaryotes coding for both classes of LysRS by the same organism. In the case of LysRS1 and LysRS2, there is evidence that the former is less sensitive to inhibitors, due to the active site of LysRS1 being more compact than that of LysRS2 [4], i.e., LysRS1 is less accommodating to lysine analogues with backbone substitutions compared with LysRS2 [5]. This bequeathed a possible selective advantage to *B. cereus *and *T. pallidum *after acquiring a copy of LysRS1: harmful lysine-analogues to LysRS in the environment bind preferentially to LysRS2, leaving LysRS1 available for unimpeded acylation of lysine to cognate tRNAs. What could be the possible selective advantage for the *Methanosarcinales *acquiring LysRS2? Comparing the rate constants (*k_cat_*) of LysRS1 and LysRS2 reveals that LysRS2 has a substrate turnover speed more than 15 times greater than that of LysRS1, while their Michaelis constants (*Km*) values are practically the same [35]. Therefore, we hypothesize that the selective advantage for coding LysRS2 is in their enhanced ability for protein synthesis. Thus, it is possible that Bacteria and Archaea coding for the two types of LysRS, in fact, developed a "safety net": in the absence of LysRS inhibitors, LysRS2 is expected to be the dominant active form. In the presence of inhibitors, LysRS1 provides a means for continuing protein synthesis.

Noteworthy, recently it was determined that in *B. cereus *LysRS1 and LysRS2 aminoacylate two tRNA species: the canonical tRNA(Lys) and a smaller RNA annotated tRNA(Other), which features a tryptophan anticodon (CCA) with a non-canonical secondary structure. tRNA(Other) was found to be synthesized only in the presence of both LysRSs, which act together during tRNA(Other) aminoacylation. This process is confined to the stationary phase, suggesting a role in growth-phase-specific protein synthesis [47].

## Conclusion

The LysRS family of enzymes has undergone several complex LGT events. The currently available data were sufficient for unambiguously identifying the origins of LysRS1 but not of LysRS2 gene transfers. The LGT transition stage of simultaneous encoding LysRS1-2 by several Archaea and Bacteria may confer a selective advantage in the presence of environmentally dependent LysRS inhibitors.

## Methods

### Data collection

LysRS1-2 sequences were retrieved from public databases: the Aminoacyl-tRNA synthetases database (aaRSDB) [19], the National Center for Biotechnology Information (NCBI) [36], the Swiss-Prot Protein knowledgebase/TrEMBL Computer-annotated supplement to Swiss-Prot [37]. 16S rRNA and 23S rRNA sequences were retrieved from the same public databases, and in addition from the Joint Genome Institute, Microbial Genomes (JGI) [38], the Ribosomal Database Project II [39] and the European Ribosomal RNA Database [40]. Additional bacterial LysRS2 sequences were obtained using NCBI Protein-protein BLAST (blastp) [41], seeded by *A. tumefaciens *LysRS2 [NCBI: NP_534951]. Additional file 1 provides a listing of the bacterial and archaeal phyla, classes and species, with corresponding LysRS2 accession numbers and their sources. Additional file 2 provides a listing of the archaeal and bacterial phyla, classes and species, with corresponding LysRS1 accession numbers and their sources.

### Reconstruction of the Archaea species tree

Species tree for 19 Archaea was based on [21,22]. It incorporates the two major phyla of the Kingdom – Crenarchaota and Euryarchaeota - and most of the representative genera in each phylum. The conspicuous exception was the absence of the psychrophilic crenarchaeon *Cenarchaeum symbiosum*. Its phylogenetic position was obtained from [42]. The tree is given in fig. 1.

### Reconstruction of the Bacteria species tree

Species tree for 43 Bacteria was based on [43], which includes the major phylogenetic relationships among phyla of the Kingdom. The phylogenetic position of most genera was obtained from the 16S rRNA based reconstruction provided in [44]. Of special interest for us were the positions of the genera within the alpha proteobacteria, because they include the site for the putative LGT event involving *A. tumeficiens *and *S. meliloti*. Specifically, the phylogenetic relationships among *A. tumeficiens*, *S. meliloti*, *B. Suis*, and *M. Loti *inferred in [44] were different depending on the gene used for the reconstruction (16S rRNA or HSP70). We therefore utilized the 23S rRNA database [39] to reconstruct a neighbor joining [46] tree of alpha-proteobacteria (with 100 bootstrap replicates) and compared the results with those given in [44]. In this reconstruction *A. tumeficiens *and *S. meliloti *clustered together with very high bootstrap support (in agreement with fig. 2b of [44]), and hence they are grouped together in fig. 2. We also utilized the 16S rRNA gene database [40] to reconstruct a neighbor joining proteobacterial phylogenetic tree with 100 replicates bootstrap, and compared our results with the trees in [23,43]; The referenced and obtained trees were in agreement (not shown).

### Reconstruction of the gene trees

Gene trees for LysRS2 and LysRS1 with bootstrap support values (100 replicates) were reconstructed using maximum likelihood (ML) as implemented in the PHYML software [48]. Among site rate variation was modeled using a gamma distribution with 4 discrete categories. Similar results were obtained using the neighbor joining reconstruction method [43] (data not shown). ML trees with bootstrap value support are presented in figs. 3 and 4, respectively. To enhance the presentation of the entire (voluminous) data, in these two figures many Bacteria and Archaea are grouped under common headings, in conformity with the presentation in the complete (unabbreviated) LysRS2 and LysRS1 ML trees, given as SM, additional files 3 and 4 respectively.

## Authors' contributions

SS and TP analyzed the data, prepared the figures and contributed to writing the manuscript, RN initiated the study and contributed to the writing of the manuscript.

## Supplementary Material

Additional file 1 Listing of the bacterial and archaeal phyla, classes and species, with the corresponding LysRS2 accession numbers and their sources Click here for file

Additional file 2 Listing of the archaeal and bacterial phyla, classes and species, with corresponding LysRS1 accession numbers and their sources. Click here for file

Additional file 3 Complete PHYML LysRS2 Maximum Likelihood gene tree in Newick format. Click here for file

Additional file 4 Complete PHYML LysRS1 Maximum Likelihood gene tree in Newick format. Click here for file
