# Supplementary material for: Paths of lateral gene transfer of lysyl-aminoacyl-tRNA synthetases with a unique evolutionary transition stage of prokaryotes coding for class I and II varieties by the same organisms
Source: BMC Evol Biol. 2006 Mar 12;6:22. doi: 10.1186/1471-2148-6-22 (PMC1475646; doi:10.1186/1471-2148-6-22)
Supplement: Additional file 1 — Listing of the bacterial and archaeal phyla, classes and species, with the corresponding LysRS2 accession numbers and their sources [file 1471-2148-6-22-S1.doc]

**SM: Additional file 1 LysRS2 - gene tree**

**The order of presentation of the data matches the order in Figure 3 and its legend in the article**

_______________________________________________________________________________________________

**Phylum Class Species Source LysRS2**

**Accession Nr.**

**__________________________________________________________________**

Crenarchaeota Thermoprotei Sulfolobus solfataricus NCBI P95970

Crenarchaeota Thermoprotei Sulfolobus tokodaii NCBI BAB67182

Crenarchaeota Thermoprotei Pyrobaculum aerophilum Swiss-Prot Q8ZWD4

--------------------------------------------------------------------------------------------------------------------------------------------

Clamidiae Clamidiae Chlamydia muridarum NCBI AAF39039 (6)

Clamidiae Clamidiae Chlamydia trachomatis NCBI O84786

Clamidiae Clamidiae Chlamydophila caviae NCBI AAP05580

Clamidiae Clamidiae Chlamydophila abortus NCBI CAH64249

Clamidiae Clamidiae Chlamydophila pneumoniae NCBI AAD19069

Clamidiae Clamidiae Parachlamydia sp NCBI CAF23952

--------------------------------------------------------------------------------------------------------------------------------------------

Proteobacteria d-proteobacteria Bdellovibrio bacteriovorus Swiss-Prot Q6MKP9

--------------------------------------------------------------------------------------------------------------------------------------------

Dein. -thermus Deinococci Thermus thermophilus NCBI P41255

Dein. -thermus Deinococci Deinococcus radiodurans Swiss-Prot Q9RXE1

-------------------------------------------------------------------------------------------------------------------------------------------

Aquificae Aquificae Aquifex aeolicus NCBI O67258

[Euryarchaeota](http://www.ncbi.nlm.nih.gov/Taxonomy/Browser/wwwtax.cgi?mode=Undef&id=28890&lvl=3&lin=f&keep=1&srchmode=1&unlock) [Methanomicrobia](http://www.ncbi.nlm.nih.gov/Taxonomy/Browser/wwwtax.cgi?mode=Undef&id=224756&lvl=3&lin=f&keep=1&srchmode=1&unlock) Methanosarcina acetivorans Swiss-Prot Q8TSN5

[Euryarchaeota](http://www.ncbi.nlm.nih.gov/Taxonomy/Browser/wwwtax.cgi?mode=Undef&id=28890&lvl=3&lin=f&keep=1&srchmode=1&unlock) [Methanomicrobia](http://www.ncbi.nlm.nih.gov/Taxonomy/Browser/wwwtax.cgi?mode=Undef&id=224756&lvl=3&lin=f&keep=1&srchmode=1&unlock) Methanosarcina mazei Swiss-Prot Q8PVP6

[Euryarchaeota](http://www.ncbi.nlm.nih.gov/Taxonomy/Browser/wwwtax.cgi?mode=Undef&id=28890&lvl=3&lin=f&keep=1&srchmode=1&unlock) [Methanomicrobia](http://www.ncbi.nlm.nih.gov/Taxonomy/Browser/wwwtax.cgi?mode=Undef&id=224756&lvl=3&lin=f&keep=1&srchmode=1&unlock) Methanosarcina barkeri Swiss-Prot Q9C4B9

-------------------------------------------------------------------------------------------------------------------------------------------

Thermotogae Thermotogae Thermotoga maritima Swiss-Prot Q9X231

--------------------------------------------------------------------------------------------------------------------------------------------

Firmicutes Clostridia Clostridium acetobutylicum Swiss-Prot Q97EB7 (55)

Firmicutes Clostridia Clostridium perfringens NCBI BAB82171

Firmicutes Clostridia Thermoanaerobacter tengcongensis Swiss-Prot Q8R7N1

Firmicutes Bacilli Bacillus licheniformis NCBI AAU21730

Firmicutes Bacilli Bacillus subtilis NCBI BAA05316

Firmicutes Bacilli Bacillus cereus Swiss-Prot Q63HC2

Firmicutes Bacilli Bacillus anthracis NCBI AAP24131

Firmicutes Bacilli Geobacillus kaustophilus Swiss-Prot Q5L440

Firmicutes Bacilli Geobacillus stearothermophilus NCBI BAA88691

Firmicutes Bacilli Bacillus halodurans Swiss-Prot Q9KGG4

Firmicutes Bacilli Bacillus clausii NCBI BAD62660

Firmicutes Bacilli Staphylococcus haemolyticus NCBI BAE05802

Firmicutes Bacilli Staphylococcus epidermidis NCBI AAO05908

Firmicutes Bacilli Staphylococcus aureus Swiss-Prot Q5HIF7

Firmicutes Bacilli Oceanobacillus iheyensis Swiss-Prot Q8EU10

Firmicutes Bacilli Exiguobacterium sp NCBI EAM86789

Firmicutes Bacilli Listeria monocytogenes Swiss-Prot Q724I4

Firmicutes Bacilli Listeria innocua Swiss-Prot Q92F47

Firmicutes Molicutes Mycoplasma pneumoniae NCBI AAB96206

Firmicutes Molicutes Mycoplasma genitalium NCBI P47382

Firmicutes Molicutes Mycoplasma gallisepticum NCBI AAP56425

Firmicutes Molicutes Ureaplasma urealyticum NCBI AAF30467

Firmicutes Molicutes Mycoplasma mycoides NCBI CAE76722

Firmicutes Molicutes Mesoplasma florum Swiss-Prot Q6F287

Firmicutes Molicutes Mycoplasma synoviae NCBI AAZ44018

Firmicutes Molicutes Mycoplasma fermentans NCBI AAC43988

Firmicutes Molicutes Mycoplasma pulmonis NCBI CAC13563

Firmicutes Molicutes Mycoplasma hominis NCBI S69892

Firmicutes Molicutes Mycoplasma mobile NCBI AAT27530

Firmicutes Molicutes Mycoplasma hyopneumoniae NCBI AAZ44294

Firmicutes Molicutes Onion.yellows phytoplasma NCBI BAD04674

Firmicutes Bacilli Streptococcus thermophilus NCBI ZP_00388129

Firmicutes Bacilli Streptococcus pneumoniae Swiss-Prot Q97RS9

Firmicutes Bacilli Streptococcus agalactiae NCBI AAM99637

Firmicutes Bacilli Streptococcus mutans NCBI AAN58493

Firmicutes Bacilli Lactococcus lactis Swiss-Prot Q9CII7

Firmicutes Bacilli Enterococcus faecalis Swiss-Prot Q839A8

Firmicutes Bacilli Lactobacillus plantarum Swiss-Prot Q88Z28

Firmicutes Bacilli Pediococcus pentosaceus NCBI ZP_00323784

Firmicutes Bacilli Lactobacillus casei NCBI ZP_00384083

Firmicutes Bacilli Leuconostoc mesenteroides NCBI ZP_00064363

Firmicutes Bacilli Oenococcus oeni NCBI ZP_00319903

Fusobacteria Fusobacteria Fusobacterium nucleatum Swiss-Prot Q8RG52

Firmicutes Clostridia Desulfitobacterium hafniense NCBI EAM94676

Firmicutes Clostridia Syntrophomonas wolfei NCBI EAO22861

Bacteroidetes Bacteroidetes Bacteroides thetaiotaomicron NCBI AAO77229

Bacteroidetes Bacteroidetes Bacteroides fragilis NCBI CAH09283

Bacteroidetes Bacteroidetes Cytophaga hutchinsonii NCBI ZP_00310321

[Chlorobi](http://www.ncbi.nlm.nih.gov/Taxonomy/Browser/wwwtax.cgi?mode=Undef&id=1090&lvl=3&lin=f&keep=1&srchmode=1&unlock) [Chlorobia](http://www.ncbi.nlm.nih.gov/Taxonomy/Browser/wwwtax.cgi?mode=Undef&id=191410&lvl=3&lin=f&keep=1&srchmode=1&unlock) Chlorobium limicola NCBI EAM42982

[Chlorobi](http://www.ncbi.nlm.nih.gov/Taxonomy/Browser/wwwtax.cgi?mode=Undef&id=1090&lvl=3&lin=f&keep=1&srchmode=1&unlock) [Chlorobia](http://www.ncbi.nlm.nih.gov/Taxonomy/Browser/wwwtax.cgi?mode=Undef&id=191410&lvl=3&lin=f&keep=1&srchmode=1&unlock) Chlorobium phaeobacteroides NCBI EAM34364

[Chlorobi](http://www.ncbi.nlm.nih.gov/Taxonomy/Browser/wwwtax.cgi?mode=Undef&id=1090&lvl=3&lin=f&keep=1&srchmode=1&unlock) [Chlorobia](http://www.ncbi.nlm.nih.gov/Taxonomy/Browser/wwwtax.cgi?mode=Undef&id=191410&lvl=3&lin=f&keep=1&srchmode=1&unlock) Prosthecochloris vibrioformis NCBI EAO14908

[Chlorobi](http://www.ncbi.nlm.nih.gov/Taxonomy/Browser/wwwtax.cgi?mode=Undef&id=1090&lvl=3&lin=f&keep=1&srchmode=1&unlock) [Chlorobia](http://www.ncbi.nlm.nih.gov/Taxonomy/Browser/wwwtax.cgi?mode=Undef&id=191410&lvl=3&lin=f&keep=1&srchmode=1&unlock) Pelodictyon **phaeoclathratiforme NCBI EAN25096**

[Chlorobi](http://www.ncbi.nlm.nih.gov/Taxonomy/Browser/wwwtax.cgi?mode=Undef&id=1090&lvl=3&lin=f&keep=1&srchmode=1&unlock) [Chlorobia](http://www.ncbi.nlm.nih.gov/Taxonomy/Browser/wwwtax.cgi?mode=Undef&id=191410&lvl=3&lin=f&keep=1&srchmode=1&unlock) Prosthecochloris aestuarii NCB IEAN21753

[Chlorobi](http://www.ncbi.nlm.nih.gov/Taxonomy/Browser/wwwtax.cgi?mode=Undef&id=1090&lvl=3&lin=f&keep=1&srchmode=1&unlock) [Chlorobia](http://www.ncbi.nlm.nih.gov/Taxonomy/Browser/wwwtax.cgi?mode=Undef&id=191410&lvl=3&lin=f&keep=1&srchmode=1&unlock) Chlorobium tepidum NCBI AAM72616

Spirochaetes Spirochaetes Leptospira interrogans Swiss-Prot Q8F4P5

-------------------------------------------------------------------------------------------------------------------------------------------

[Planctomycetes](http://www.ncbi.nlm.nih.gov/Taxonomy/Browser/wwwtax.cgi?mode=Undef&id=203682&lvl=3&lin=f&keep=1&srchmode=1&unlock) [Planctomycetacia](http://www.ncbi.nlm.nih.gov/Taxonomy/Browser/wwwtax.cgi?mode=Undef&id=203683&lvl=3&lin=f&keep=1&srchmode=1&unlock) Rhodopirellula baltica NCBI CAD77044

Proteobacteria a-proteobacteria Agrobacterium tumefaciens NCBI NP_534951

Proteobacteria a-proteobacteria Sinorhizobium meliloti NCBI CAC47483

[Chloroflexi](http://www.ncbi.nlm.nih.gov/Taxonomy/Browser/wwwtax.cgi?mode=Undef&id=200795&lvl=3&lin=f&keep=1&srchmode=1&unlock) [Dehalococcoidetes](http://www.ncbi.nlm.nih.gov/Taxonomy/Browser/wwwtax.cgi?mode=Undef&id=301297&lvl=3&lin=f&keep=1&srchmode=1&unlock) Dehalococcoides ethenogenes NCBI AAW40144

----------------------------------------------------------------------------------------------------------------------------------------- ---

Acidobacteria Acidobacteria Solibacter usitatus NCBI EAM57887 (3)

Proteobacteria d-proteobacteria Anaeromyxobacter dehalogenans NCBI EAL80760

Actinobacteria Actinobacteria Symbiobacterium thermophilum Swiss-Prot Q67S33

---------------------------------------------------------------------------------------------------------------------------------------------

[Cyanobacteria](http://www.ncbi.nlm.nih.gov/Taxonomy/Browser/wwwtax.cgi?mode=Undef&id=1117&lvl=3&lin=f&keep=1&srchmode=1&unlock) [Nostocales](http://www.ncbi.nlm.nih.gov/Taxonomy/Browser/wwwtax.cgi?mode=Undef&id=1161&lvl=3&lin=f&keep=1&srchmode=1&unlock) Anabaena variabilis NCBI ZP_00161636 (9)

[Cyanobacteria](http://www.ncbi.nlm.nih.gov/Taxonomy/Browser/wwwtax.cgi?mode=Undef&id=1117&lvl=3&lin=f&keep=1&srchmode=1&unlock) [Nostocales](http://www.ncbi.nlm.nih.gov/Taxonomy/Browser/wwwtax.cgi?mode=Undef&id=1161&lvl=3&lin=f&keep=1&srchmode=1&unlock) Nostoc sp NCBI BAB75770

[Cyanobacteria](http://www.ncbi.nlm.nih.gov/Taxonomy/Browser/wwwtax.cgi?mode=Undef&id=1117&lvl=3&lin=f&keep=1&srchmode=1&unlock) [Chroococcales](http://www.ncbi.nlm.nih.gov/Taxonomy/Browser/wwwtax.cgi?mode=Undef&id=1118&lvl=3&lin=f&keep=1&srchmode=1&unlock) Synechococcus elongates NCBI ZP_00164084

[Cyanobacteria](http://www.ncbi.nlm.nih.gov/Taxonomy/Browser/wwwtax.cgi?mode=Undef&id=1117&lvl=3&lin=f&keep=1&srchmode=1&unlock) [Chroococcales](http://www.ncbi.nlm.nih.gov/Taxonomy/Browser/wwwtax.cgi?mode=Undef&id=1118&lvl=3&lin=f&keep=1&srchmode=1&unlock) Synechocystis sp NCBI P73443

[Cyanobacteria](http://www.ncbi.nlm.nih.gov/Taxonomy/Browser/wwwtax.cgi?mode=Undef&id=1117&lvl=3&lin=f&keep=1&srchmode=1&unlock) [Chroococcales](http://www.ncbi.nlm.nih.gov/Taxonomy/Browser/wwwtax.cgi?mode=Undef&id=1118&lvl=3&lin=f&keep=1&srchmode=1&unlock) Thermosynechococcus longatus NCBI BAC07765

[Cyanobacteria](http://www.ncbi.nlm.nih.gov/Taxonomy/Browser/wwwtax.cgi?mode=Undef&id=1117&lvl=3&lin=f&keep=1&srchmode=1&unlock) [Prochlorales](http://www.ncbi.nlm.nih.gov/Taxonomy/Browser/wwwtax.cgi?mode=Undef&id=1212&lvl=3&lin=f&keep=1&srchmode=1&unlock) Prochlorococcus marinus Swiss-Prot Q7UZP0

[Cyanobacteria](http://www.ncbi.nlm.nih.gov/Taxonomy/Browser/wwwtax.cgi?mode=Undef&id=1117&lvl=3&lin=f&keep=1&srchmode=1&unlock) [Chroococcales](http://www.ncbi.nlm.nih.gov/Taxonomy/Browser/wwwtax.cgi?mode=Undef&id=1118&lvl=3&lin=f&keep=1&srchmode=1&unlock) Synechococcus sp Swiss-Prot Q5N1D9

[Cyanobacteria](http://www.ncbi.nlm.nih.gov/Taxonomy/Browser/wwwtax.cgi?mode=Undef&id=1117&lvl=3&lin=f&keep=1&srchmode=1&unlock) [Gloeobacteria](http://www.ncbi.nlm.nih.gov/Taxonomy/Browser/wwwtax.cgi?mode=Undef&id=307596&lvl=3&lin=f&keep=1&srchmode=1&unlock) Gloeobacter violaceus Swiss-Prot Q7NG18

[Cyanobacteria](http://www.ncbi.nlm.nih.gov/Taxonomy/Browser/wwwtax.cgi?mode=Undef&id=1117&lvl=3&lin=f&keep=1&srchmode=1&unlock) [Oscillatoriales](http://www.ncbi.nlm.nih.gov/Taxonomy/Browser/wwwtax.cgi?mode=Undef&id=1150&lvl=3&lin=f&keep=1&srchmode=1&unlock) [Trichodesmium erythraeum](http://www.ncbi.nlm.nih.gov/Taxonomy/Browser/wwwtax.cgi?id=1206) NCBI EAO28784

--------------------------------------------------------------------------------------------------------------------------------------------

Actinobacteria Actinobacteria Leifsonia xyli NCBI AAT89834 (15)

Actinobacteria Actinobacteria Brevibacterium linens NCBI ZP_00378892

Actinobacteria Actinobacteria Corynebacterium efficiens NCBI BAC19343

Actinobacteria Actinobacteria Corynebacterium glutamicum NCBI NP_601883

Actinobacteria Actinobacteria Corynebacterium jeikeium Swiss-Prot Q4JXL1

Actinobacteria Actinobacteria Mycobacterium avium NCBI AAS02776

Actinobacteria Actinobacteria Mycobacterium tuberculosis NCBI O06284

Actinobacteria Actinobacteria Mycobacterium leprae NCBI P46861

Actinobacteria Actinobacteria Nocardia farcinica NCBI BAD55253

Actinobacteria Actinobacteria Bifidobacterium longum NCBI AAN25440

Actinobacteria Actinobacteria Arthrobacter sp NCBI EAL97549

Actinobacteria Actinobacteria Propionibacterium acnes NCBI AAT81939

Actinobacteria Actinobacteria Nocardioides sp NCBI EAO06074

Actinobacteria Actinobacteria Thermobifida fusca NCBI ZP_00293151

Actinobacteria Actinobacteria Frankia sp NCBI EAN14067

---------------------------------------------------------------------------------------------------------------------------------------------

Proteobacteria d-proteobacteria Desulfovibrio desulfuricans NCBI ZP_00131094 (80)

Proteobacteria d-proteobacteria Desulfovibrio vulgaris NCBI AAS96849

Proteobacteria d-proteobacteria Syntrophobacter fumaroxidans NCBI EAO20646

Proteobacteria d-proteobacteria Desulfotalea psychrophila NCBI CAG36358

Proteobacteria d-proteobacteria Geobacter sulfurreducens Swiss-Prot Q74AT0

Proteobacteria d-proteobacteria Geobacter metallireducens NCBI EAM78203

Proteobacteria d-proteobacteria Pelobacter propionicus NCBI EAO35297

Proteobacteria d-proteobacteria Desulfuromonas acetoxidans NCBI EAM69994

Proteobacteria e-proteobacteria Helicobacter hepaticus NCBI AAP78263

Proteobacteria e-proteobacteria Helicobacter pylori NCBI AAD07251

Proteobacteria e-proteobacteria Wolinella succinogenes Swiss-Prot Q7MAR1

Proteobacteria e-proteobacteria Campylobacter coli NCBI EAL56722

Proteobacteria e-proteobacteria Campylobacter jejuni Swiss-Prot Q5HW66

Proteobacteria e-proteobacteria Campylobacter upsaliensis NCBI EAL52475

Proteobacteria e-proteobacteria Campylobacter lari NCBI EAL54226

Proteobacteria e-proteobacteria Thiomicrospira denitrificans NCBI EAO04113

Proteobacteria unclassified Uncultured Magnetococcus sp NCBI EAN28803

Proteobacteria g-proteobacteria Xanthomonas oryzae NCBI AAW76126

Proteobacteria g-proteobacteria Xanthomonas axonopodis NCBI AAM36738

Proteobacteria g-proteobacteria Xylella fastidiosa Swiss-Prot Q87EB3

Environmental sample Uncultured bacterium 580 NCBI AAS07872

Proteobacteria b-proteobacteria Neisseria gonorrhoeae NCBI AAW90095

Proteobacteria b-proteobacteria Neisseria meningitides Swiss-Prot Q9JTT7

Proteobacteria b-proteobacteria Chromobacterium violaceum Swiss-Prot Q7NZ62

Proteobacteria b-proteobacteria Azoarcus sp NCBI CAI10033

Proteobacteria b-proteobacteria Dechloromonas aromatica NCBI ZP_00151178

Proteobacteria b-proteobacteria Burkholderia cepacia NCBI ZP_00212730

Proteobacteria b-proteobacteria Burkholderia cenocepacia NCBI EAM12821

Proteobacteria b-proteobacteria Burkholderia vietnamiensis NCBI EAM29872

Proteobacteria b-proteobacteria Burkholderia mallei NCBI AAU47811

Proteobacteria b-proteobacteria Burkholderia fungorum NCBI ZP_00283787

Proteobacteria b-proteobacteria Ralstonia eutropha NCBI ZP_00170910

Proteobacteria b-proteobacteria Ralstonia metallidurans NCBI EAN53144

Proteobacteria b-proteobacteria Ralstonia solanacearum NCBI CAD14730

Proteobacteria b-proteobacteria Polaromonas sp NCBI EAM41395

Proteobacteria b-proteobacteria Bordetella pertussis NCBI CAE41400

Proteobacteria b-proteobacteria Bordetella bronchiseptica NCBI CAE32789

Proteobacteria b-proteobacteria Bordetella parapertussis NCBI CAE37345

Proteobacteria b-proteobacteria Methylobacillus flagellatus NCBI EAN03032

Proteobacteria b-proteobacteria Nitrosomonas eutropha NCBI EAO17616

Proteobacteria b-proteobacteria Nitrosomonas europaea Swiss-Prot Q82SH1

Environmental sample Uncultured bacterium 577 NCBI AAR38056

Proteobacteria b-proteobacteria Thiobacillus denitrificans NCBI ZP_00335928

Proteobacteria g-proteobacteria Methylococcus capsulatus NCBI AAU90453

Proteobacteria g-proteobacteria Idiomarina loihiensis Swiss-Prot Q5QYV9

Proteobacteria g-proteobacteria Coxiella burnetii NCBI AAO89981

Proteobacteria g-proteobacteria Legionella pneumophila Swiss-Prot Q5WVS0

Proteobacteria g-proteobacteria Francisella tularensis Swiss-Prot Q5NI89

Proteobacteria g-proteobacteria Wigglesworthia glossinidia NCBI BAC24587

Proteobacteria g-proteobacteria Buchnera aphidicola NCBI BAB13135

Proteobacteria g-proteobacteria Candidatus Blochmannia pennsyl. NCBI AAZ40901

Proteobacteria g-proteobacteria Escherichia coli NCBI CAD66193

Proteobacteria g-proteobacteria Citrobacter freundii NCBI AAP40013

Proteobacteria g-proteobacteria Shigella flexneri Swiss-Prot Q83JU6

Proteobacteria g-proteobacteria Salmonella typhi Swiss-Prot Q8Z3X8

Proteobacteria g-proteobacteria E.coli heat/induced Swiss-Prot Q8FAT5

Proteobacteria g-proteobacteria Yersinia pestis Swiss-Prot Q8ZHK5

Proteobacteria g-proteobacteria Photorhabdus luminescens Swiss-Prot Q7N1C8f

Proteobacteria g-proteobacteria Erwinia carotovora Swiss-Prot Q6D945

Proteobacteria g-proteobacteria Pasteurella multocida NCBI P57822

Proteobacteria g-proteobacteria Haemophilus somnus NCBI ZP_00131887

Proteobacteria g-proteobacteria Haemophilus influenzae NCBI P43825

Proteobacteria g-proteobacteria Pseudomonas putida NCBI AAN67117

Proteobacteria g-proteobacteria Pseudomonas aeruginosa NCBI ZP_00137095

Proteobacteria g-proteobacteria Pseudomonas siringae NCBI AAZ33422

Proteobacteria g-proteobacteria Azotobacter vinelandii NCBI EAM05680

Proteobacteria g-proteobacteria Psychrobacter cryohalolentis NCBI EAO10955

Proteobacteria g-proteobacteria Psychrobacter arcticum NCBI AAZ18906

Proteobacteria g-proteobacteria Acinetobacter sp NCBI CAA86924

Proteobacteria g-proteobacteria Chromohalobacter salexigens NCBI EAM22372

Proteobacteria g-proteobacteria Microbulbifer degradans NCBI ZP_00315209

Proteobacteria g-proteobacteria Shewanella oneidensis NCBI AAN54065

Proteobacteria g-proteobacteria Shewanella baltica NCBI EAN44386

Proteobacteria g-proteobacteria Shewanella denitrificans NCBI EAN72165

Proteobacteria g-proteobacteria Shewanella frigidimarina NCBI EAN75347

Proteobacteria g-proteobacteria Shewanella amazonensis NCBI EAM57887

Proteobacteria g-proteobacteria Vibrio vulnificus NCBI BAC93433

Proteobacteria g-proteobacteria Vibrio cholerae NCBI AAF93829

Proteobacteria g-proteobacteria Vibrio fischeri NCBI AAW84948

Proteobacteria g-proteobacteria Colwellia psychrerythraea NCBI AAZ26689

------------------------------------------------------------------------------------------------------------------------------------------
