# Supplementary material for: Paths of lateral gene transfer of lysyl-aminoacyl-tRNA synthetases with a unique evolutionary transition stage of prokaryotes coding for class I and II varieties by the same organisms
Source: BMC Evol Biol. 2006 Mar 12;6:22. doi: 10.1186/1471-2148-6-22 (PMC1475646; doi:10.1186/1471-2148-6-22)
Supplement: Additional file 2 — Listing of the archaeal and bacterial phyla, classes and species, with corresponding LysRS1 accession numbers and their sources. [file 1471-2148-6-22-S2.doc]

**SM: Additional file 2 LysRS1 – gene tree**

**The order of presentation of the data matches the order in Figure 4 and its legend in the article**

________________________________________________________________________________________________

**Phylum Class Species Source LysRS1**

**Accession Nr.**

**__________________________________________________________________**

Crenarchaea Thermoprotei Cenarchaeum symbiosum NCBI O74059

------------------------------------------------------------------------------------------------------------------------------------------------

Proteobacteria a-proteobacteria Sphingomonas aromaticivorans NCBI ZP_00094269 **(11)**

Proteobacteria a-proteobacteria Wolbachia sp. Swiss-Prot Q73GS2

Proteobacteria a-proteobacteria Rickettsia conorii Swiss-Prot Q92IB5

Proteobacteria a-proteobacteria Rickettsia prowazekii Swiss-Prot Q9ZDF8

Proteobacteria a-proteobacteria Magnetospirillum magnetotacticum NCBI ZP_00054350

Proteobacteria a-proteobacteria Rhodopseudomonas palustris NCBI CAE30125

Proteobacteria a-proteobacteria Caulobacter crescentus Swiss-Prot Q9ABY6

Proteobacteria a-proteobacteria Rhodobacter sphaeroides NCBI ZP_00005479

Proteobacteria a-proteobacteria Mesorhizobium loti Swiss-Prot Q98BC8

Proteobacteria a-proteobacteria Brusella suis NCBI P59225

Proteobacteria a-proteobacteria Brucella melitensis Swiss-Prot Q8YCM8

------------------------------------------------------------------------------------------------------------------------------------------------

Crenarchaea Thermoprotei Aeropyrum pernix Swiss-Prot Q9YFT9

------------------------------------------------------------------------------------------------------------------------------------------------

Euryarchaeota Halobacteria Halobacterium sp. Swiss-Prot Q9HNN7

------------------------------------------------------------------------------------------------------------------------------------------------

Euryarchaeota Methanopyri Methanopyrus kandleri Swiss-Prot Q8TWP6 (11)

Euryarchaeota Methanobacteria Methanobacterium thermoautotrophicum NCBI O27585

Euryarchaeota Thermoplasmata Ferroplasma acidarmanus NCBI ZP_00001413

Euryarchaeota Thermoplasmata Thermoplasma acidophilum NCBI P57677

Euryarchaeota Thermoplasmata Thermoplasma volcanium Swiss-Prot Q979B8

Euryarchaeota Archaeoglobi Archaeoglobus fulgidus NCBI O29052

Euryarchaeota Methanomicrobia Methanosarcina barkeri Swiss-Prot Q9C4B7

Euryarchaeota Methanomicrobia Methanosarcina acetivorans Swiss-Prot Q8TTA3

Euryarchaeota Methanomicrobia Methanosarcina mazei Swiss-Prot Q8PWA

Euryarchaeota Methanococci Methanococcus maripaludis NCBI O30522

Euryarchaeota Methanococci Methanococcus jannaschii Swiss-Prot Q57959

----------------------------------------------------------------------------------------------------------------------------------------------

Euryarchaeota Thermococci Pyrococcus furiosus Swiss-Prot Q8U4F3

Euryarchaeota Thermococci Pyrococcus abyssi Swiss-Prot Q9V270

Euryarchaeota Thermococci Pyrococcus horikoshii NCBI O57963

----------------------------------------------------------------------------------------------------------------------------------------------

Spirochaeetes Spirochaeetes Borrelia burgdorferi NCBI O51603

Spirochaeetes Spirochaeetes Treponema denticola Swiss-Prot Q73JY6

Spirochaeetes Spirochaeetes Treponema pallidum NCBI O83650

----------------------------------------------------------------------------------------------------------------------------------------------

Firmicutes Bacilli Bacillus cereus NCBI NP_829979

---------------------------------------------------------------------------------------------------------------------------------------------

Actinobacteria Actinobacteria Streptomyces avermitilis Swiss-Prot Q82E67

Actinobacteria Actinobacteria Streptomyces coelicolor Swiss-Prot Q9X895

---------------------------------------------------------------------------------------------------------------------------------------------
